# Interface‐Induced Stability of Nontrivial Topological Spin Textures: Unveiling Room‐Temperature Hopfions and Skyrmions

**DOI:** 10.1002/adma.202511754

**Published:** 2025-08-18

**Authors:** Ferhat Katmis, Valeria Lauter, Rawana Yagan, Iuri S. Brandt, Arash M. Cheghabouri, Hua Zhou, John W. Freeland, Clodoaldo I. L. de Araujo, Michelle E. Jamer, Don Heiman, Mehmet C. Onbasli, Jagadeesh S. Moodera

**Affiliations:** ^1^ Department of Physics Massachusetts Institute of Technology Cambridge MA 02139 USA; ^2^ Francis Bitter Magnet Laboratory & Plasma Science and Fusion Center Massachusetts Institute of Technology Cambridge MA 02139 USA; ^3^ Neutron Scattering Division Neutron Sciences Directorate Oak Ridge National Laboratory Oak Ridge TN 37831 USA; ^4^ Department of Electrical and Electronics Engineering Koç University Istanbul 34450 Türkiye; ^5^ Departamento de Física Universidade Federal de Viçosa Viçosa 36570‐900 Brazil; ^6^ Advanced Photon Source Argonne National Laboratory Argonne IL 60439 USA; ^7^ Physics Department United States Naval Academy Annapolis MD 21402 USA; ^8^ Department of Physics Northeastern University Boston MA 02115 USA; ^9^ Department of Physics Koç University Istanbul 34450 Türkiye

**Keywords:** hopfions, micromagnetic simulations, skyrmions, spin textures, topological insulators

## Abstract

Topological spin configurations, such as soliton‐like spin texture and Dirac electron assemblies, have recently emerged in fundamental science and technology. Achieving stable topological spin textures at room temperature is crucial for their use as long‐range information carriers. However, their creation and manipulation are hindered by multi‐step field training and competing interactions. Thus, a spontaneous ground state for multidimensional topological spin textures is desirable, with skyrmions forming swirling, hedgehog‐like spin structures in two dimensions and hopfions as their twisted 3D counterparts. Here, the first observation of robust and reproducible topological spin textures of hopfions and skyrmions observed at room temperature and in zero magnetic field is reported, which are stabilized by geometric confinement and protected by interfacial magnetism in a ferromagnet/topological insulator/ferromagnet trilayer heterostructure. These skyrmion‐hopfion configurations are directly observed at room temperature with Lorenz transmission electron microscopy. Using micromagnetic modeling, the experimental observations of hopfion‐skyrmion assemblies are reproduced. This model reveals a complete picture of how spontaneously organized skyrmion lattices encircled by hopfion rings are controlled by surface electrons, uniaxial anisotropy, and Dzyaloshinskii‐Moriya interaction. This study provides evidence that topological chiral spin textures can facilitate the development of magnetic topological carriers, paving the way for ultralow‐power and high‐density information processing.

## Graphical abstract

The first room‐temperature, zero‐field observation of stable skyrmion–hopfion spin textures in EuS─Bi_2_Se_3_─EuS trilayers is reported. Combining Lorentz TEM imaging and micromagnetic modeling, the authors unveil how interfacial Dzyaloshinskii–Moriya interaction and geometric confinement stabilize these multidimensional topological states. These findings offer a pathway toward ultralow‐power spintronic devices harnessing robust, chiral magnetism in engineered topological heterostructures.

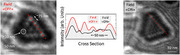

## 1 Introduction

In order to minimize both size and power dissipation in future electronics, using spin degrees of freedom rather than strictly electronic charge, a durable and robust remanence will be required against external perturbation.^[^ ^1^, ^2^ ^]^ In addition, the desired stability to thermal fluctuations at room temperature may be achieved by topological protection of the states encoded in chiral spins. Thanks to their intriguing quantum features, topological insulators (TIs) hold immense promise for advancing technological applications to new heights.^[^ ^3^, ^4^, ^5^, ^6^, ^7^, ^8^, ^9^, ^10^, ^11^, ^12^, ^13^, ^14^, ^15^, ^16^, ^17^ ^]^ When the ferromagnetic insulator (FMI) that surrounds the TI acquires a nonuniform magnetization, the interplay between Dirac electrons and the domain wall gives rise to a chiral state.^[^ ^18^, ^19^, ^20^ ^]^ A unique interfacial chiral spin texture can be stabilized via coupling surface states with magnetic anisotropy thus bringing new functionalities to TI systems.^[^ ^21^, ^22^, ^23^, ^24^, ^25^, ^26^, ^27^, ^28^, ^29^, ^30^, ^31^ ^]^

Dissipation‐free topologically protected surfaces of TIs serve as a unique platform for the formation of skyrmion–hopfion assemblies.^[^ ^32^, ^33^, ^34^, ^35^ ^]^ Similar to 2D skyrmions, hopfions are soliton‐like topologically protected spin textures that have been theoretically discovered and empirically confirmed in different material systems.^[^ ^35^, ^36^, ^37^, ^38^, ^39^, ^40^, ^41^, ^42^, ^43^, ^44^, ^45^, ^46^ ^]^ The origin of such localized helical magnetic textures is the competition between the Dzyaloshinskii–Moriya interaction (DMI) favoring non‐collinearity and the Heisenberg exchange interaction favoring collinear alignments.^[^ ^45^, ^46^, ^47^, ^48^, ^49^, ^50^, ^51^, ^52^, ^53^ ^]^ Achieving stable, topological multi‐dimensional soliton‐like spin textures and Dirac electron assemblies at room temperature remains challenging due to multi‐step field training methods and competing interactions. To enable their use as long‐range and dissipation‐free information carriers at nanoscale, a spontaneous stable ground state for these textures is highly desirable.

We report the first observation of hopfion–skyrmion configuration at room temperature under zero applied magnetic field in a trilayer FMI‐TI‐FMI heterostructure of EuS─Bi_2_Se_3_─EuS thin film. Remarkably, these hopfion–skyrmion assemblies are observed far above the bulk Curie temperature of EuS, ≈17 K. The chiral nature is driven by the interfacial DMI between adjacent FMI and TI, while the coupling strength at the two interfaces provides a tunable non‐collinear spin texture as well as skyrmion lattice and hopfions. Through extensive characterizations and imaging, it is demonstrated that the chiral spin configuration exists at ambient temperature, even though the total magnetic moment reduces beyond the Curie temperature, evidently enabled by the strong DMI and interfacial coupling. Finally, the entire trilayer structure undergoes a phase transition between a ferromagnet and a unique combination of skyrmion and hopfion phase, which is confirmed by real space observation using a Lorentz transmission electron microscope (LTEM).

## 2 Results and Discussion

Magnetically coupled EuS─Bi_2_Se_3_─EuS hybrid trilayer structures were grown using a well‐optimized epitaxial technique.^[^ ^8^ ^]^ The trilayer films were grown simultaneously on silicon nitride (Si_3_N_4_) membranes and on sapphire substrates to study the spatially‐ and depth‐resolved magnetic configuration; further information is provided in the Experimental Section.

**Figure 1:**
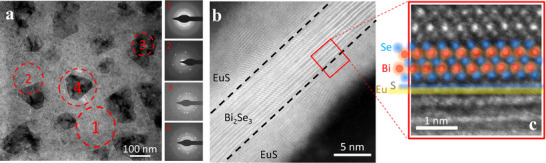
a,b) High‐resolution TEM measurements for trilayer EuS─Bi_2_Se_3_─EuS (5 nm–5 QL–5 nm) grown on a Si_3_N_4_ membrane. In (a), the HRTEM top‐view image of a large area of a sample grown on a grid. The dark regions are single crystalline domains surrounded by polycrystalline lighter region, which are labeled as Region 1. A single domain island is labeled as Region 4. A closer look at a crystalline domain, where two domains merge to form a twin boundary labeled as Region 3. Selected area electron diffraction patterns of the corresponding regions (1, 2, 3, and 4) are shown between (a) and (b). In (b), a cross‐sectional bright‐field TEM image of the trilayer sample on a membrane is shown, and an associated enlarged image of the highlighted region of one interface is indicative of well‐oriented large crystallites in **c**.

We performed high‐resolution LTEM, which enables lateral magnetization mapping,^[^ ^54^, ^55^ ^]^ while the film quality was surveyed with conventional high‐resolution transmission electron microscopy (HRTEM) techniques. The top view and cross‐sectional view are shown in **Figure** **1**a–c, respectively; the spatial atomic distribution of a trilayer is shown in Figure S9 (Supporting Information). The initial EuS layer deposited on the membrane exhibits a textured structure that serves as an effective seed layer for the subsequent growth of TI. This layer reduces the interface energy, promoting the 2D growth of the TI layer. Over time, the TI layer evolves through the merging of smaller domains into larger domains, such coalescence resulting in either twin domains or a single expanded domain. To distinguish such domains, we labeled the regions; Region 1 is identified for polycrystalline phases and is confirmed by a broad ring electron diffraction pattern. Region 3 was taken on a twin domain. The cross‐section cut shown in Figure 1b was made along a single‐domain island as in Region 4. The trilayer configuration is clearly evident in the cross‐section view. The area that has been highlighted reveals sharp interfaces between each atomic layer, as shown in Figure 1c. The position of the atoms, the van der Waals gap, and the EuS monolayers are all straightforwardly distinct. The interface between the EuS and Bi_2_Se_3_ layers is clearly defined, with an abrupt interface transition without chemical interdiffusion, according to the atomic density profile fitting. Typically, a weak van der Waals connection forms at the interfacial region between the S and Se layers located directly on top of each other with a typical gap size of 2.45 ± 0.1 Å, which is confirmed by an X‐ray diffraction analysis for the layers grown on sapphire (See Figure S1, Supporting Information).

**Figure 2:**
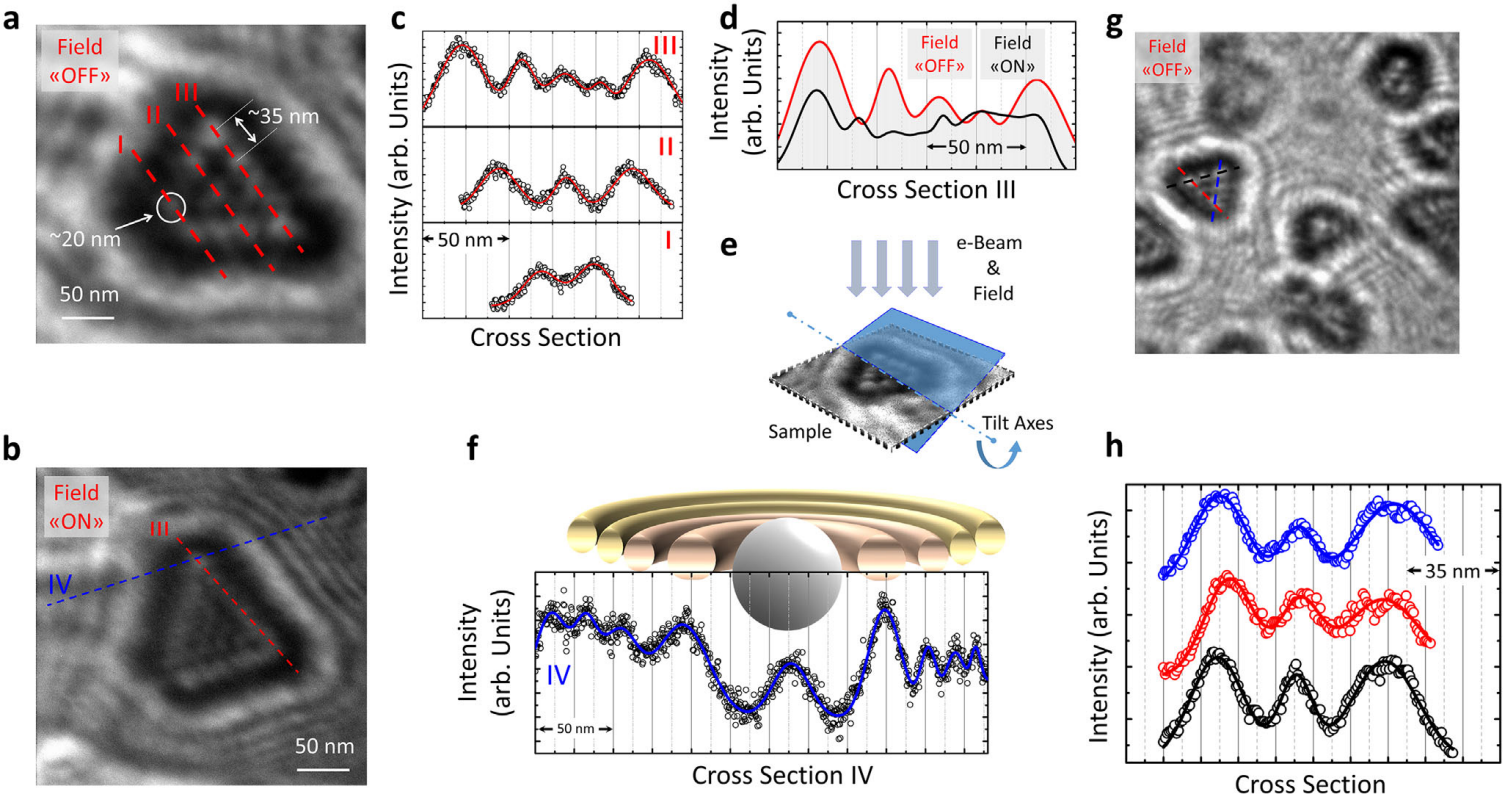
a–h) Lorentz TEM measurements at room temperature for trilayer EuS─Bi_2_Se_3_─EuS (5 nm–5 QL–5 nm) grown on Si_3_N_4_ membrane. The LTEM was performed when the field is ON in (a), and OFF in (b) at room temperature. A closer look at one of the truncated islands, where the triangular skyrmion lattice is formed with a periodicity of 35 ± 5 nm with the size of each skyrmion 20 ± 5 nm. In (c), the distinct skyrmion lattice line cut is seen along I, II, and III in (a). In (d), the magnetic field applied out‐of‐plane by the microscope objective lens when the sample is tilted shows an increase in the density of skyrmion in the lattice as in the histogram data. The applied out‐of‐plane component of the magnetic field is 1.53 T, “Field ON” condition. The periodicity is shown as the integrated intensity histogram of the lateral cross‐sectional along III without field (in red) and with field (in black). In (e), a schematic illustration of the imaging configuration while manipulating the sample under a magnetic field. In (f), standing wave patterns of spins outside of the crystal region are plotted along the line intersecting IV in (b). These peaks demonstrate the presence of skyrmion lattice points at the edge, along with the hopfion ring. Additionally, clear spin wave formations surrounding the island exhibit wave‐like characteristics that are clearly visible in (b). Above it is the schematic graphic that serves as a guide for the eyes. In (g), a closer look at one of the smaller size triangular islands where the perfect triangular lattice is formed with the same periodicity. The integrated intensity histogram for the island seen in (g) is displayed along each of the three side edges in (h).

LTEM experiments were carried out on single crystalline domains of different sizes at room temperature, shown in **Figure** **2**. In a zero magnetic field, we observe skyrmions arranged in a triangular‐symmetric lattice with bright spots of 20±5 nm diameter at the vertices of the triangles and are spaced 35±5 nm apart from each other in Figure 2c. This type of spontaneous ordering is associated with the deflection of the electron beam due to the features of the curled magnetization of skyrmion core.^[^ ^36^, ^56^ ^]^ Observing ring like features, attributed to hopfion rings, surrounding the lattices of triangularly structured skyrmions is another significant finding.

It is remarkable that the dynamics and stability of skyrmions and hopfions may be influenced by one another. We observed that when an out‐of‐plane magnetic field is applied, the density of skyrmions increases, and hopfions become more organized inside each island, as shown in Figure 2a,b. From the figure, it is estimated that increasing the applied field decreases the skyrmion diameter down to ≈10 nm before Heisenberg exchange‐limited length‐scale regime is reached, which is consistent with previously observed skyrmion sizes.^[^ ^7^, ^10^ ^]^ The value of the saturation field depends on the size of the island and can be estimated as 1 Tesla or more, which exceeds the LTEM bias capability. By examining different islands, it was found that the diameter of the skyrmions and the nature of the lattice formation were similar on different islands.

As can be seen in Figure 1, the trilayer creates crystalline domain islands, and between the islands there is a region with polycrystalline characteristics, which has a lower damping nature than the crystalline region. This magnetic and structural confinement opens the way for nucleation of hopfions at the boundaries of the islands. Owing to the strong magnetic properties of solitons, they were found to also act on the polycrystalline region, where standing wave patterns of spins are generated as a result of collective spin excitation.^[^ ^57^ ^]^ As can be seen in Figure 2f and the Figure S2 (Supporting Information) showing how standing wave patterns of spins propagate with the magnetic field, the periodicity of the standing waves and the hopfion diameter decreases with increasing perpendicular field as in Figure S2b (Supporting Information). Furthermore, as can be seen in Figure 2g with linear cuts in 2 h along the three edges, we observe the interference pattern of standing wave patterns of spins excitations at a long‐range scale in such independent islands (Figure S2, Supporting Information). The height profile analysis confirms that the observed magnetic phenomena are intrinsic to the EuS/Bi_2_Se_3_/EuS heterostructure, with line cuts along the red and black dashed lines in Figure S2c–e (Supporting Information) revealing the characteristic truncated island morphology of epitaxially grown Bi_2_Se_3_ exhibiting thickness variations of ≈2 QLs between adjacent structural units. LTEM demonstrates that both the magnetic contrast and the emergent hopfion–skyrmion assemblies maintain structural and magnetic coherence across islands despite these thickness fluctuations.

Our systematic investigation across various thickness combinations (Bi_2_Se_3_: 10–20 nm; EuS: 1–10 nm) demonstrates that interfacial spin textures remain qualitatively consistent, with the EuS thickness primarily affecting overall magnetic moment while maintaining constant interfacial exchange coupling strength per unit area. The magnetic proximity effect decays exponentially into Bi_2_Se_3_ with a characteristic length scale of ≈1–2 nm, confining the induced spin texture to interface regions and allowing the opposite surface to approach intrinsic helical Dirac states beyond ≈6–8 nm thickness. Laterally, Bi_2_Se_3_ forms truncated islands of 50–200 nm that serve as fundamental structural units, with magnetic domains (100–500 nm) creating scenarios where individual islands may contain single domains or span domain boundaries, resulting in intriguing multi‐domain configurations. This island‐limited geometry provides an advantageous framework for studying topological surface state interactions with magnetic proximity effects across both structural and magnetic boundaries while maintaining consistent behavior within homogeneous regions.

To investigate the long‐range magnetic order, continuous epitaxial trilayer EuS─Bi_2_Se_3_─EuS films were grown on a single crystal *c*‐plane sapphire (Al_2_O_3_(0001)) substrate, see Experimental Section for details. In epitaxial trilayers, bulk volume‐ and surface‐sensitive techniques were used to confirm the existence of sharp chemical and electronic interfaces, as has been used in the case of bilayer samples.^[^ ^8^ ^]^ The bottom and top EuS layers have six‐fold symmetry due to the nature of the rotating domain, either on the substrates or on Bi_2_Se_3_, respectively. From the structural investigation as shown in Figure S1 (Supporting Information), the bottom‐up crystalline symmetry of the top EuS makes both layers structurally identical.

**Figure 3:**
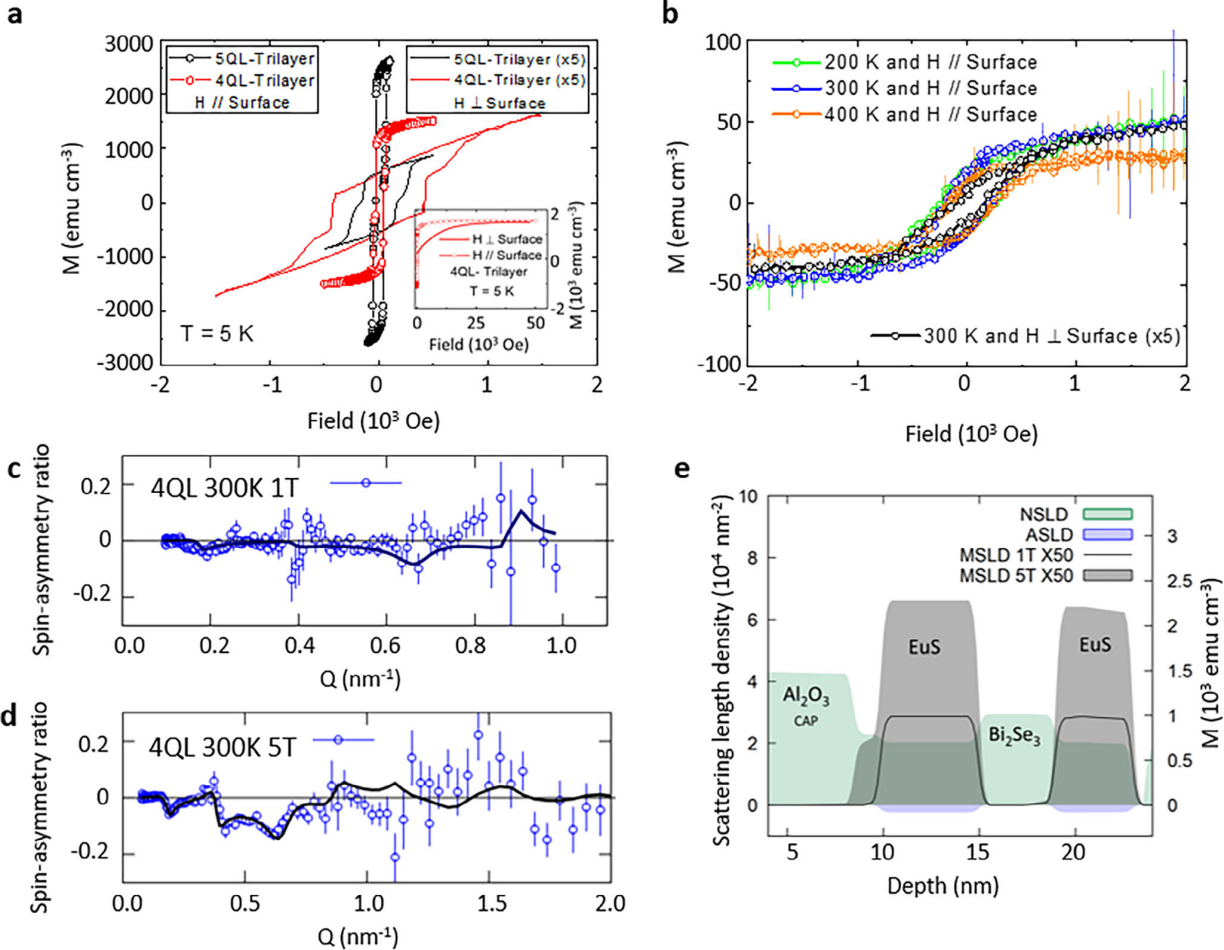
a–e) Superconducting quantum interference device (SQUID) magnetometry and PNR measurements for trilayer EuS─Bi_2_Se_3_─EuS, In (a), measurements of **M**(**H**) at low field and temperature in a parallel and perpendicular field configuration for 4 QL‐trilayer (EuS (5 nm)─Bi_2_Se_3_ (4 QL)─EuS (5 nm)) and 5 QL‐trilayer (EuS (5 nm)─Bi_2_Se_3_ (5 QL)─EuS (5 nm)) samples. The **M**(**H**) at high field region for a 4‐QL trilayer sample with parallel and perpendicular arrangement is displayed in the inset. (b) shows the **M**(**H**) for the 5 QL trilayer at high temperatures in parallel and perpendicular field configurations. The corresponding spin‐asymmetry (SA) ratio and model fits shown by solid lines, *SA* = (*R* ^+^ − *R* ^−^)/(*R* ^+^ + *R* ^−^), derived from the simultaneous fitting of the polarized neutron reflectivity measurement shown for 4 QL‐trilayer samples under 1 T in (c) and 5 T in‐plane field in (d). (e) displays neutron nuclear (NSLD, green), magnetic (MSLD, grey), and absorption (ASLD, purple) scattering length density profiles across the trilayer epitaxial sample which were recorded at 300 K with in‐plane field of 1 and 5 T.

Although proximity‐induced magnetic phenomena are apparent from the macroscopic magnetization measurements (see for example, **Figure** **3**a,b), the contribution of interfacial magnetism between EuS and Bi_2_Se_3_ at low temperatures is small compared to the bulk magnetism of EuS. Interfacial effects at high temperatures become more apparent when bulk magnetism mostly vanishes above the Curie temperature.^[^ ^8^ ^]^ Element‐specific X‐ray absorption (XAS) and X‐ray magnetic circular dichroism (XMCD) measurements were performed using total electron yield detection mode, which provides enhanced surface/interface sensitivity compared to bulk fluorescence yield. XMCD spectra for the Eu^2+^ state as a function of temperature show a sign of significant interfacial coupling, as presented in Figure S10 (Supporting Information). While XMCD at Se L‐edges showed magnetic dichroism, spectral overlap with one of the Eu edges prevents unambiguous attribution to Se magnetization. The observed interfacial magnetic proximity effects are confirmed through complementary polarized neutron reflectometry analysis. It is crucial to emphasize that due to the highly localized nature of the exchange interaction, achieving an exceptionally sharp, well‐defined, and clean interface between EuS and TI is imperative to ensure the necessary effective magnetic proximity coupling. Therefore, careful handling is essential during their formation and subsequent magnetic interfacial structuring in order to minimize any unwanted contamination from leftover chalcogen atoms.^[^ ^58^ ^]^

For elucidating the spin texture and the spacial distribution of magnetism in the trilayer films, we employed the depth‐sensitive polarized neutron reflectometry (PNR) to reveal the non‐collinear magnetic order along the two interacting heterointerfaces. In the case of bilayer samples, the ferromagnetism at 5K extends to about 2 nm into the TI layer, while due to such short‐range nature the time‐reversal symmetry could only be broken in the vicinity of the interfacial region.^[^ ^8^ ^]^ When an additional magnetic interface is created near to the other interface, close enough to interact, the total magnetic configuration changes dramatically. To overcome the anisotropy, the PNR reflectivity profiles R^+^ and R^−^ were measured at 300 K in saturating in‐plane external magnetic field of 1 and 5 T. PNR results reveal that the two coupled interfaces make both EuS layers in FMI‐TI‐FMI trilayer totally magnetic to much higher temperatures, the corresponding depth profiles of neutron structural, magnetic and absorption scattering length density (NSLD as green, MSLD as grey, and ASLD as purple) are shown in Figure 3e for 300 K, and 5 K in Figure S3 (Supporting Information). The corresponding spin asymmetry (SA) data are shown in Figure 3c,d for 1 and 5 T, respectively. As an indicator of Eu atoms, the ASLD depth profile^[^ ^59^ ^]^ ends right at the Bi_2_Se_3_ interface, indicating that no Eu atoms were found in the Bi_2_Se_3_ layer. Consequently, PNR provides concrete proof that these trilayer heterostructure exhibits room‐temperature ferromagnetism produced by proximity. Depth‐sensitive PNR reveals that in tri‐layer EuS/Bi_2_Se_3_/EuS heterostructures, both EuS layers maintain magnetization throughout their entire 5 nm thickness up to 300 K, representing a significant enhancement over bi‐layer samples where magnetization was retained only in a narrow ≈2 monolayer of EuS interfacial region. This suggests that the sandwiched Bi_2_Se_3_ layer acts as a magnetic mediator, extending interface‐driven magnetic stabilization through long‐range coupling mechanisms, with the double‐interface geometry providing stronger magnetic coupling than conventional bi‐layer configurations.

Using micromagnetic models based on the Landau–Lifshitz–Gilbert (LLG) formalism and aided by the LTEM, SQUID, and PNR data, the origin of protected chiral magnetism at various temperatures has been examined for the occurrence of the magnetic texture skyrmion–hopfion assembly (See Experimental Section for details in the Supporting Information). Experimental observations of the chiral spins were closely reproduced using micromagnetic modeling and their numerical solutions. Phase diagrams of hopfion with skyrmion lattice under different geometric confinement have been generated in a broad parameter space, such as different DMI settings, uniaxial anisotropy parameters, and saturation magnetizations.

The effects of changes in the DMI and saturation magnetization *M*_sat_ on the stability of a single skyrmion island are illustrated in Figure S4 (Supporting Information), where the initial settings without hopfion are displayed. The results can be categorized into three areas: 1) A skyrmion with an out‐of‐plane magnetic orientation for lower *M*_sat_ and lower DMI can occur in certain areas. 2) A skyrmion might still be stabilized under different magnetic parameter combinations and increased *M*_sat_ for the in‐plane film magnetization. 3) The increased DMI dominates the system and produces a labyrinth‐like magnetism because of the decreased anisotropy and exchange energy. Note that the parameter window *M*_sat_ and DMI that stabilizes the hopfion with skyrmion lattice in the models are consistent with the earlier experimental results.^[^ ^8^ ^]^

**Figure 4:**
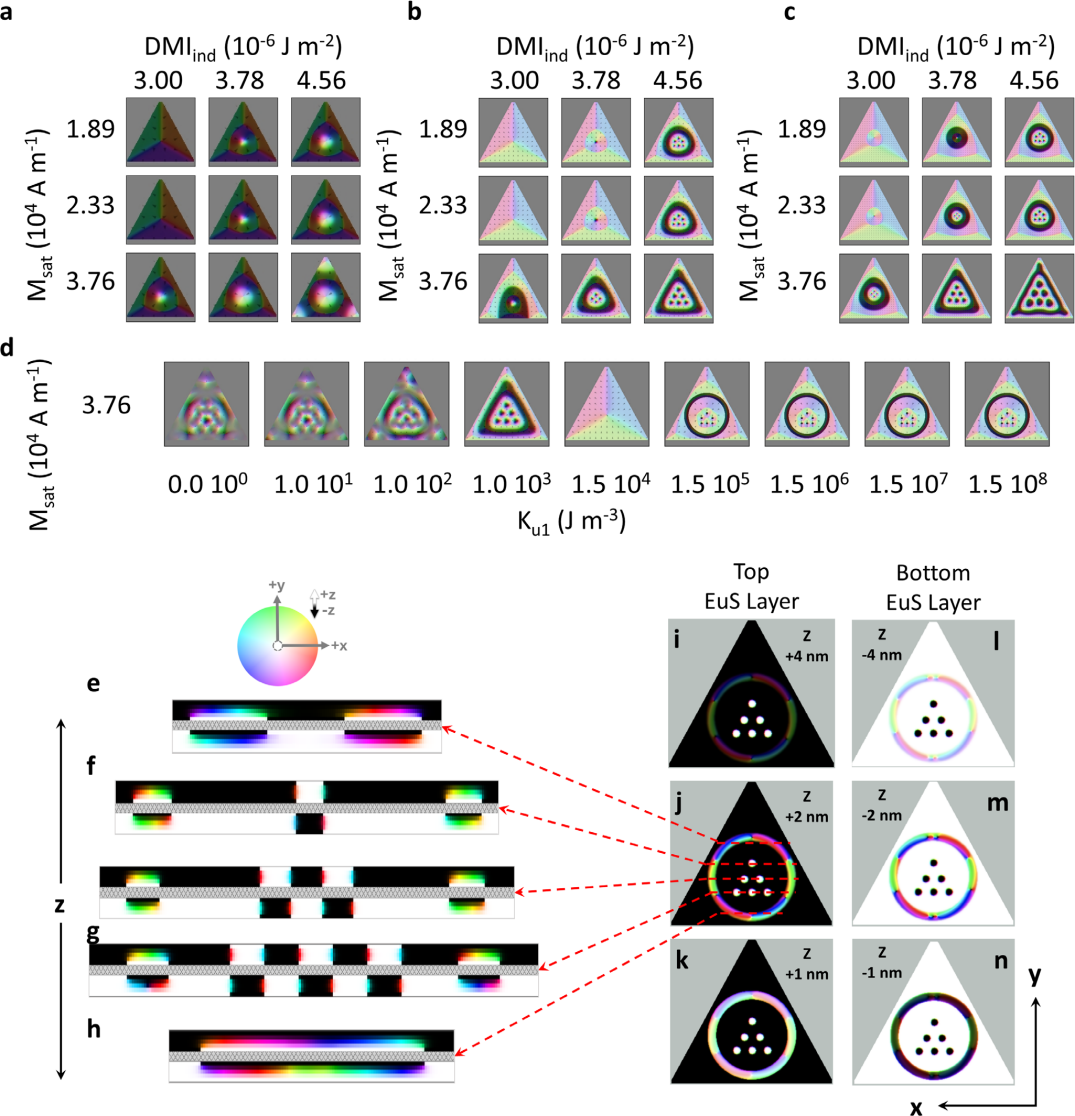
a–n) Micromagnetic simulations of magnetic texture with hopfion rings for trilayer films. The corresponding simulations for Figure 2g were run on regular triangle geometry with sizes at 100 in (a), 300 in (b), and 500 nm^2^ in (c). The entire isosurfaces of the skyrmion and hopfion lattice construction are matched with experimental data in (b), due to their accurate size and shape by convenient *M*_sat_ and DMI parameters. Skyrmion lattice surrounded by a hopfion ring initialized in a uniform domain of +**m** (white) in a triangular geometry as features of ‐**m** (black) and ran a simulation to obtain a final relaxed output. In (d), a large range was also run for the corresponding uniaxial anisotropy parameters *K*_u1_ with DMI_ind_ = 4.56 × 10^−6^ J m^−2^, *A*_ex_ = 1.94 × 10^−14^ J m^−1^, and *M*_sat_ = 37.6 kA m^−1^ with regular triangle geometry with sizes at 300 nm^2^. The phase profile along the *x*–*z* plane at different *y* positions is displayed in (e–h), while the lateral view is depicted in the *x*–*y* plane is shown in (i–n). Lateral cross‐sections were extracted at different EuS thicknesses, specifically at ±1, ±2, and ±4 nm, corresponding to their distances from each Bi_2_Se_3_ interface, where the + and – signs indicate positions within the top and bottom EuS layers, respectively. The color bar represents the spin phase changes corresponding to increasing azimuthal and polar angles.

**Figure** **4** illustrates how the formation of skyrmion and hopfion rings are influenced by the geometrical confinement, with island sizes of 100 (Figure 4a), 300 (Figure 4b), and 500 nm^2^ (Figure 4c). The geometrical size defines the scale of the hopfion and skyrmion lattice. This imposes strong demagnetization fields, and the symmetry is broken at very small geometric sizes, as in case of the 100 nm island, in Figure 4a, due to competition between the edges and magnetic features. Furthermore, after relaxation of the model, the influence of the DMI constant and the saturation magnetization on the stability of the hopfion ring protecting the skyrmion lattice is demonstrated. The ring diffuses into the triangle geometry's borders at low DMI and *M*_sat_, and the magnetic features' topology is lost. The hopfion ring remains unbroken at higher DMI and *M*_sat_, as particularly seen at DMI = 4.56 × 10^−6^ J m^−2^ and *M*_sat_ = 37.6 kA m^−1^. This is also demonstrated for the reduced number of lattice‐forming skyrmions, shown in the Figure S6 (Supporting Information), similar to the observed features in the experimental data of Figure 2g. By rigorously checking the derived parameters during both the coarse (Figure S7, Supporting Information) and fine (Figure S8, Supporting Information) evolution phases, a comprehensive analysis of the entire parameter space was performed. This study revealed a relatively narrow operating regime that favors the formation of skyrmion–hopfion assemblies.

From our micromagnetic analysis, the ring in‐plane diameter is ≈21 nm, and each skyrmion has a diameter of 20–25 nm for various numbers of confined skyrmions and independent of geometries as in the experimental observation. These results suggest that the fingerprint of the topological magnetic ordering is unique for the geometric confinement and intrinsic magnetic parameters. For zero or low uniaxial anisotropy constant *K*_u1_ (0–10^2^ J m^−3^), the magnetic features exhibit a diffused ground state as shown in Figure 4d. When *K*_u1_ is increased to 10^3^ J m^−3^ in the out‐of‐plane direction (+**z**), the magnetic topological order exhibits the skyrmion–hopfion assembly that resembles the LTEM results shown in Figure 2g. In the high *K*_u1_ regime (10^5^–10^8^ J m^−3^), the uniaxial anisotropy energy term starts dominating over the other magnetization terms including DMI, demagnetization, or geometry effects, intralayer exchange (*A*_ex_), interlayer exchange, and Ruderman–Kittel–Kasuya–Yoshida (RKKY) interaction. The hopfion and the skyrmion lattice could be stabilized in the micromagnetic models when the exchange, in‐plane demagnetization, and the DMI fields are selected.

Previous studies suggested a similar stabilization mechanism in which RKKY,^[^ ^60^ ^]^ the Bloembergen–Rowland interaction,^[^ ^61^ ^]^ spin‐orbit coupling,^[^ ^62^ ^]^ and antiferromagnetic coupling on the surface help to stabilize the interface magnetism.^[^ ^63^ ^]^ Micromagnetic models show that antiferromagnetic chiral ordering is stabilized for both interfaces at the same time when the RKKY energy term is included. The fact that an RKKY term is needed for the stability implies that the magnetism at both EuS‐Bi_2_Se_3_ interfaces is likely coupled. The phase diagrams provide evidence that the stable magnetic topological ordered features could be achieved under the explored material parameter space, as shown in Figure S11 (Supporting Information).

Our micromagnetic simulations predict Néel‐type skyrmions in the ultra‐thin film limits, and we have ensured that the simulation closely match those of the experimental conditions.^[^ ^51^, ^55^, ^64^ ^]^ The Fresnel oscillations that are observed consistently in both our simulations and experiments align with the expected features for Néel‐type skyrmions, especially in the presence of interfacial DMI. We cannot observe bulk/Bloch skyrmions to be stabilized in ultra‐thin film limit as in phase profile analysis Figure 4e–n. Once the thickness increases to 20 nm EuS, we show that bulk/Bloch skyrmions are obtained in our micromagnetic models as shown in Figure S12 (Supporting Information). The skyrmion phase profile observed in Figure S12 (Supporting Information) is consistent with the definition of the bulk/Bloch skyrmion.^[^ ^65^ ^]^

Our analysis reveals topological features consistent with hopfions, clearly distinguishing them from skyrmion bags. Specifically, our system consists of ultra‐thin layers, only a few nanometers thick, where DMI arises exclusively from interfacial effects. The dimensional confinement in our system gives rise to unique magnetic configurations that differ significantly from those observed in bulk‐like materials.^[^ ^66^ ^]^ The simulated and analyzed structures are governed by this interfacial DMI and strong in‐plane demagnetizing fields, resulting in the confinement of the spin phase profile of hopfions to within a few unit cells. To clarify, we have performed a detailed 3D analysis of the magnetic textures in our simulations, and a thorough investigation of the magnetization profiles along all spatial dimensions as shown in Figure 4 and Figure S12 (Supporting Information). These analyses clearly demonstrate that the observed structures possess a non‐trivial topology, which is characteristic of hopfions, rather than mere stacking of skyrmion bags.

A systematic comparison of EuS/Bi_2_Se_3_/EuS heterostructures and bulk‐like FeGe systems reveals significant differences in the demagnetizing field effects due to the ultrathin nature of the films. In contrast to bulk FeGe,^[^ ^33^ ^]^ these nanometer‐scale structures exhibit significantly modified demagnetizing fields. Analysis of radial, azimuthal, and polar spin phase profiles in heterostructures with 20 and 5 nm thick EuS layers confirms the presence of fiber‐like nontrivial topological configurations, including hopfion rings and skyrmions. Increasing the film thickness reduces the strong in‐plane demagnetizing fields, facilitating appearance of swirling or fiber‐like topological textures of hopfions, distinctly different from those in bulk FeGe. The dimensional confinement in these thin films induces unique magnetic configurations, governed by the interfacial DMI and strong in‐plane demagnetizing fields, which confine the spin phase profile of the hopfions to a few unit cells. This extreme spatial confinement challenges the visual reconstruction of hopfion geometries compared to the more flexible formation in bulk FeGe, highlighting the critical influence of interfacial and confinement effects on topological spin textures.

The interface coupling also depends on the initial configuration, whereas the magnetic topological ordered structures are stable once initialized in the forms indicated in the phase diagrams. Despite the large in‐plane demagnetizing field in the nanoscale geometric confinement of the irregular shape trilayers in the remanent state, the robust stability of the hopfion with skyrmion lattice at room temperature reported in other studies^[^ ^67^, ^68^ ^]^ suggests that these additional energy terms will be necessary to maintain the equilibrium lattice at room temperature. It has been discovered that, for the parameter choices considered in the models, the topological order is unstable at room temperature. Reliable prediction of magnetic order at room temperature is hampered by the uncertainty in magnetic parameters such as DMI, RKKY, and other uniaxial or strain‐related anisotropy constants, as well as the crucial role of surface states. Nevertheless, the room temperature magnetic order can be partially reproduced when the uniaxial anisotropy parameter is on the order of 10^7^ and 10^8^ J m^−3^.

The geometric confinement identified in our study enables room‐temperature zero‐field stability by providing a controlled platform to demonstrate the underlying interfacial phenomena. This confinement reveals the fundamental stabilization mechanisms governing topological spin textures at ferromagnet–TI interfaces, where long‐range magnetic ordering extends throughout the entire ferromagnetic layer thickness. Our findings illuminate the fundamental physics governing topological structure stabilization, providing pathways to engineer wider area interfacial systems through alternative approaches such as interfacial engineering or strain modulation that replicate the beneficial effects of geometric confinement.

The first‐principles study^[^ ^63^ ^]^ identified magnetic proximity effects in trilayer EuS/Bi_2_Se_3_/EuS systems, demonstrating exponential decay of interlayer exchange with topological insulator thickness. While the concept of hidden DMI suggests opposite‐sign interfacial DMI at top and bottom interfaces due to inversion symmetry,^[^ ^69^, ^70^ ^]^ experimental validation shows DMI is intrinsically short‐range and localized within 1–2 nm of FMI/TI interfaces.^[^ ^11^, ^71^ ^]^ Given our ≈5 nm Bi_2_Se_3_ spacer thickness, the interfacial DMI fields at opposite surfaces remain effectively independent without direct coupling, while interface‐level asymmetries from domain boundaries and crystallographic imperfections naturally break the inversion symmetry conditions required for hidden DMI cancellation.

## 3 Conclusion

These findings demonstrate the capability to generate multi‐dimensional spin textures, such as hopfions and skyrmions, on topological insulator surfaces through precise control of interfacial coupling and geometric confinement. The combination of experimental observations and micromagnetic modeling reveals that interfacial Dzyaloshinskii–Moriya interactions and spin‐momentum locking at the topological insulator interface are key to stabilizing these textures at room temperature. These spin structures potentially enable low‐dissipation information manipulation, which could, with further engineering advances in electrode design and operating conditions, eventually contribute to more energy‐efficient data storage and transmission technologies. The ability to engineer stable spin textures through interface design offers a pathway to integrate these topologically protected states into spintronic devices, where they could enable low‐power, high‐speed operations. Moreover, the potential interaction of these textures with quantum states, such as Majorana fermions, opens avenues for topological quantum computing. The robustness of these structures at ambient temperatures positions them as promising candidates for practical applications in next‐generation memory, logic, and sensing devices.

## 4 Experimental Section

### Material Growth

The epitaxial growth of both trilayers (EuS─Bi_2_Se_3_─EuS) grown on sapphire and membrane were performed simultaneously. The entire process was carried out in a custom‐built molecular beam epitaxy setup equipped with an electron beam evaporator for EuS deposition and effusion cells with 5 n (99.999%) purity Bi and Se at a base pressure of 2 × 10^−10^ Torr. As a final stage, all multilayers were protected in situ with amorphous Al_2_O_3_ layers. From the bottom up, each interface formation and structural evolution were monitored by an in situ reflection high‐energy electron diffraction apparatus. Due to the high reactivity of Eu atoms and dissociation problems of S, the initial EuS was evaporated congruently from a single electron‐beam source at 260 ± 5 °C with a rate of 1.0–1.5 Å s^−1^. After the first EuS layer growth, the temperature of the effusion cells was increased to prevent Se incorporation into the layer. The temperature of the substrate was maintained at 240 ± 5 °C, while the temperature of the cells was raised; this procedure takes ≈1 h. Bi and Se were then co‐evaporated with a flux ratio of 1:15 A growth rate of 1–2 Å min^−1^ was utilized to create an ultra‐smooth surface in order to prevent kinetic surface roughening. The temperature of the effusion cells was lowered immediately after Bi_2_Se_3_ growth. The growth stage was maintained at growth temperature during the cooling down process of the cells. As the temperature of the cells dropped, the second EuS layer was deposited with the same growth methodology as the initial layer. Finally, in the same deposition chamber, 5–10 nm amorphous Al_2_O_3_ was deposited in situ as a protective layer to all trilayer films. An energy dispersive spectroscopy (EDS) map shows the atomic distribution for a trilayer sample grown on the Si_3_N_4_ membrane. Based on the element mapping as shown in Figure S9 (Supporting Information), the elements Bi, Se, Eu, and S were uniformly distributed across the film surface.

### Structural Analysis

Crystal‐truncation rod (CTR) measurements analyzed by coherent Bragg rod analysis (COBRA) method was used to render the real space electron density profile across the interfacial area with atomic accuracy, in an effort to shed light on the interface microstructure.^[^ ^72^ ^]^ The CTR measurements were carried out at the Beamline 12‐ID‐D of Advanced Photon Source (APS), Argonne, using a six‐circle diffractometer with an X‐ray energy of 20 keV (wavelength *λ* = 0.6199 Å) beam. Using the COBRA approach, the sample's specular CTR (00L) for both the bilayer and trilayer film configurations was examined. The interface between EuS and Bi_2_Se_3_ layers, as well as between Bi_2_Se_3_ and sapphire single crystal, were clearly delineated from the total electron density profile. Resolving the interface bond clearly demonstrates a sharp interface transition in the absence of any chemical inter‐diffusion. Se atoms were in direct contact with either the S layer on EuS or the last oxygen layer of sapphire for bilayer films. Bi_2_Se_3_ was terminated at each interface by the Se layer, and the S layer was directly stacked on top of the Se layer by interfacial van der Waals bonding spacing of 2.45 Å. Overall, an excellent epitaxial cube‐on‐hexagon growth connection was seen for EuS (111) on Bi_2_Se_3_ and sapphire.

*SQUID magnetometry measurements* were taken to observe the magnetization state of the multilayer systems and performed in a quantum design MPMS superconducting quantum interference device (SQUID) magnetometer. Both in‐plane and out‐of‐plane magnetic properties were measured in the temperature range of 5–400 K and applied magnetic fields up to 5 T.

*Lorentz Microscopy measurements* ^[^ ^73^ ^]^ were performed on samples grown on silicon nitride grids at room temperature. The good quality of trilayer structure was achieved in well‐defined grains. From the TEM images it is possible to note regular arrays of helical magnetic structures with lattice parameter depending on the grain geometry. The Cs‐probe corrected transmission electron microscope FEI Titan Themis 80–300 kV, was employed to perform HRTEM, EDS, and LTEM using Fresnel method (out‐of‐focus). The experiments were carried out at 300 kV and RT. A special Lorentz lens allows LTEM imaging in magnetic field‐free conditions when the standard objective lens was switched off. By appropriately increasing the objective lens excitation, a magnetic field arises and was perpendicular to the sample surface. However, before exciting the objective lens, in order to apply a magnetic field to the sample, it was tilted up to 40°. For the “Field‐ON” configuration shown in Figure 2, the applied out‐of‐plane component of the magnetic field is 1.53 T. Specifically, the defocus values were 34 µm for conventional TEM imaging and from 0.89 to 1.38 mm for magnetic imaging.

Exit wave function of diffracted electrons in LTEM (non‐magnetic crystal, no external magnetic field): To capture the Fresnel oscillations of transmitted electrons in LTEM, the exit wave function of electrons were initially defined, considering the periodicity of the nonmagnetic crystal structure. Subsequently, the spin structure was incorporated, which exhibits an incommensurate periodicity relative to the reciprocal wave vector of the lattice.

The translational symmetry of the crystal brings a reciprocal lattice wave vector **G**. In a crystal with a periodic potential, *V*(**r**) =  *V*(**r** + **a**), where **a** is a lattice vector. This periodic lattice potential leads to the Bloch wave solutions with wave vector **k** and *u*_k_(**r**) = *u*_k_ (**r** + **a**); ψ(**r**) = *u*_k_ (**r**)*e* ^*i* **k** · **r**^. The exit wave function in LTEM becomes: ψ_exit_ (**r**) = ψ_incident_ (**r**)*e* ^*i*ϕ(**r**)^, where ϕ(**r**) is the phase shift introduced by the crystal potential. For a thin crystal of thickness t, ϕ(**r**) is

(1) $$\phi \;\left(r\right)=\frac{\sigma }{\hbar v}\;\int_{0}^{t}V\left(r,z\right)dz$$

Fresnel oscillations arise due to the interference of multiple scattered waves. A crystal potential with a Fourier expansion in the reciprocal lattice can be used to describe the transmitted electron wave function. Thus, the transmitted wave can be written as a sum of diffracted waves

(2) $$\psi_{\mathrm{trans}}\;\left(r\right)=\sum_{G}A_{G}e^{i\left(k+G\right)\cdot r}\;;\;I\;\;\;\left(r\right)={\left|\psi_{\mathrm{trans}}\left(r\right)\right|}^{2}={\left|\sum_{G}A_{G}e^{i\left(k+G\right)\cdot r}\right|}^{2}$$

The amplitudes of the diffracted waves *A*_**G**_ were determined by the scattering conditions, which depend on both sample and measurement parameters. Here, *I*(**r**) is the intensity observed in LTEM. This expression contains the interference terms $A_{G}A_{G^{\prime }}^{*}$ and *e* ^*i*[(**G** − **G′**) · **r**]^, which lead to the oscillatory patterns in the intensity corresponding to the LTEM Fresnel oscillations of nonmagnetic crystals.

When the spin order with wave vector **q**, was introduced, that is incommensurate with the crystal wave vector **G** and *V*_**q**_ are the Fourier components of the spin‐modulated magnetic potential: $V_{\mathrm{spin}}\;\left(r\right)=\sum_{q}V_{q}e^{iq\cdot r}$. For a magnetic crystal with spin ordering, the total potential contains the contributions from the periodic lattice potential *V*_lattice_(**r**) and the spin wave potential *V*_spin_(**r**); *V*_total_ (**r**) = *V*_lattice_ (**r**) + *V*_spin_(**r**). When external magnetic field was applied, the Schrödinger equation must be modified to include the Aharonov–Bohm phase, which includes the vector magnetic potential (∇  ×  **A**  =** **B**) that modifies the electron wavefunction's phase. The Zeeman interaction term also modifies the spin‐dependent potential

(3) $$-\frac{\hbar^{2}}{2m}{\left(\nabla -i\frac{e}{\hbar }A\right)}^{2}\psi \left(r\right)+V_{\mathrm{total}}\left(r\right)\psi \;\left(r\right)=E\psi \left(r\right)$$

Under a constant magnetic field $B=B\hat{z}$, the vector potential can be expressed in the symmetric gauge as $A\;=1/2B\times r=1/2B\;\left(-y\hat{x}+x\hat{y}\right)$. The Zeeman interaction accounts for the coupling of electron's magnetic moment to external magnetic field. This interaction modifies the different spin wave potential from *V*_spin_(**r**) to *V*_spin_(**r**) + Δ*V*_Zeeman_. The modified wave function is now subject to the total potential from the lattice, spin wave, and the Zeeman contribution: *V*_total_ (**r**) = *V*_lattice_ (**r**) + *V*_spin_(**r**) + Δ*V*_Zeeman_. The phase shift in the exit wavefunction becomes

(4) $$\phi \;\left(r\right)=\frac{\sigma }{\hbar v}\;\int_{0}^{t}V_{\mathrm{total}}\left(r,z\right)dz+\frac{e}{\hbar }\int A\cdot dr$$

The exit wave function for magnetic crystal with spin wave order under magnetic field is thus ψ_exit_ (**r**) = ψ_incident_ (**r**)*e* ^*i*ϕ(**r**)^

(5) $$\psi_{\mathrm{trans}}\;\left(r\right)=\sum_{G}A_{G}e^{i\left(k+G\right)\cdot r+i\frac{e}{\hbar }\int A\cdot dr}+\sum_{q}A_{q}e^{i\left(k+q\right)\cdot r+i\frac{e}{\hbar }\int A\cdot dr}$$

(6) $$I\left(r\right)={\left|\psi_{\mathrm{trans}}\left(r\right)\right|}^{2}={\left|e^{i\frac{e}{\hbar }\int A\cdot dr}\right|}^{2}\;{\left|\sum_{G}A_{G}e^{i\left(k+G\right)\cdot r\;}+\sum_{q}A_{q}e^{i\left(k+q\right)\cdot r\;}\right|}^{2}$$

The Aharonov–Bohm phase contribution cancels out in the intensity expression shown in the final *I*(***r***).

(7) $$I\;\left(r\right)={\left|\sum_{G}A_{G}e^{i\left(k+G\right)\cdot r\;}+\sum_{q}A_{q}e^{i\left(k+q\right)\cdot r\;}\right|}^{2}$$

The Fresnel oscillations from crystal lattice symmetry remain unchanged by an external magnetic field, while the **q**‐vector in the second summation term depends on the field due to skyrmion or spin wave dispersion. This was the only field‐sensitive component. These experiments confirmed that the external field modifies spin wave dispersion, that the observed LTEM contrast was purely a lattice effect. The field‐induced changes in spin wave dispersion altered the peak positions, phases, and intensities of Fresnel oscillations, demonstrating that the observed LTEM contrast was magnetic in origin. Thus, Fresnel fringes originated from the interplay of lattice symmetry, skyrmion structures, and spin wave dispersion.

*PNR measurements* were performed on the magnetism reflectometer at the Spallation Neutron Source at Oak Ridge National Laboratory.^[^ ^74^, ^75^, ^76^, ^77^ ^]^ Neutrons with wavelengths within a band of 2–8 Å and with a high polarization of 99% to 98.5% were used. Measurements were performed in a closed cycle refrigerator (Advanced Research System CCR) with an applied external magnetic field by using a 1.15 T Bruker electromagnet and in a top‐loading closed‐cycle refrigerator combined with a 5 T cryomagnet (Cryomagnetics). Using the time‐of‐flight method, a collimated polychromatic beam of polarized neutrons with the wavelength band Δ*λ* impinged on the film at a grazing incidence angle, *θ*, where it interacts with atomic nuclei and the spins of unpaired electrons. The reflections *R* ^+^ and *R* ^−^, were measured as a function of momentum transfer, *Q* = 4 π sin*θ*/*λ*, for two neutron polarizations with the neutron spin parallel (+) or antiparallel (−) to the direction of the external field, *H*_ext_. To separate nuclear from magnetic scattering, the data were presented as the spin‐asymmetry (SA) ratio SA = (*R* ^+^(Q) – *R* ^−^(Q))/(*R* ^+^(Q) + *R* ^−^(Q)). A value of SA = 0 indicates that there was no magnetic moment in the system. Being electrically neutral, spin‐polarized neutrons penetrated the entire multilayer structure and probed the magnetic and structural composition of the film through buried interfaces down to the substrate. PNR was a highly penetrating depth‐sensitive technique to investigate the magnetic and chemical composition of the system with 0.5 nm resolution.^[^ ^78^ ^]^ The NSLD and MSLD depth profiles corresponded to the depth distribution of the chemical and IP magnetization vector distributions at the atomic scale, respectively. Spin‐polarized neutrons at grazing incidence penetrated the entire multilayer film down to the substrate and provide depth‐resolved information about the structure and the magnetization vector of the film through buried interfaces. It was noted that neutron interactions with Eu atoms were affected by unpaired *f*‐electrons with unique magnetic properties. The distinct characteristics of Eu led to variations in the neutron absorption scattering length density (ASLD), in contrast to other elements. It was essential to closely examine the distinctive properties of materials containing Eu atoms while analyzing their neutron absorption behavior.^[^ ^59^ ^]^ To spatially resolve the net magnetization vector through the depth of trilayer configurations, the authors used a time‐of‐flight method using a highly collimated polychromatic beam of polarized neutrons impinging on the film under grazing incidence and interacting with atomic nuclei and the spins of unpaired electrons providing the information on the depth profile of the nuclear and magnetic scattering length densities (NSLD and MSLD) corresponding to chemical and in‐plane magnetization vector distributions, respectively.^[^ ^74^, ^75^, ^76^, ^77^ ^]^

*Micromagnetic simulations*, Micromagnetic models and simulation results have been proposed to explain the magnetic ordering of the trilayer system (EuS‐Bi_2_Se_3_‐EuS) shown in Figure 1 as a hopfion ring encircling the skyrmion lattice. Using MuMax3 software^[^ ^79^ ^]^ based on LLG equation, micromagnetic modeling was conducted to investigate the feasibility of constructing such a system using the following material parameters: saturation magnetization *M*_sat_ = 37.6 × 10^4^ A m^−1^, Heisenberg exchange constant *A*_ex_ = 1.94 × 10^−14^ J m^−1^, interfacial constant DMI = 4.56 × 10^−6^ J m^−2^ and Gilbert damping constant *α* = 0.3. The mesh was set to 1 × 1 × 1 nm^3^ for a 100, 300, and 500 nm^2^ geometry, with each EuS and Bi_2_Se_3_ layers having a thickness of 5 nm. For each top and bottom interface layer of EuS, *M*_sat_ was reduced to one‐third of its original value and the simulation was relaxed for the hopfion–skyrmion system.

Having a narrower hopfion ring would not survive against the edge effects and dipolar fields from the skyrmion lattice. The anisotropy field was chosen as *K*_u1_ = 1.0 × 10^6^ J m^−3^ in the out‐of‐plane (+**z**) direction. Due to this high value, the authors observed little or no relaxation of the magnetic features as this anisotropy field overcomes the demagnetization field. Hopfions were expected to show chirality in three dimensions, and the moments surrounding the ring will swirl around it;^[^ ^80^ ^]^ however, due to the high uniaxial anisotropy field, the authors did not observe these effects (Figures S7 and S8, Supporting Information). The width of the hopfion kept it intact even in proximity to the skyrmion lattice, and despite the strong demagnetization or dipolar interactions between them. If the magnetic features were allowed to relax further, one could expect the hopfion to lose its symmetric shape and the shape of its domain walls extending to the edge of the geometry due to edge interactions (Figure S8, Supporting Information). The modeling identified the material parameter ranges (*M*_sat_ and DMI) needed for stabilizing the skyrmion lattice with and without the hopfion ring. These ranges are presented in Figures S4 and S5 (Supporting Information) for irregular hexagon islands.

### XMCD

The samples were examined using XMCD to verify interfacial magnetic coupling. At the Advanced Photon Source's beamline 4‐ID‐C, a number of soft X‐ray absorption spectroscopy investigations were conducted by simultaneously measuring the bulk‐sensitive fluorescence yield and the surface‐sensitive total electron yield. Initially, the magnetic condition of the EuS layer was investigated. With a valence state of 2+, the EuS layer exhibited a significant XMCD, and its lineshape was in line with a local moment of 7 *µ*_B_ per atom. Figure S10 (Supporting Information) displays the XMCD spectra for the Eu^2+^ state as a function of photon energy as a function of temperature to obtain the magnetic properties of Eu atoms after the moments were aligned by an applied perpendicular 5 T field training. The scans shown were averaged over many scans and analyzed with X‐ray energy over the Eu *M*_5_‐edge (≈1128 eV, 3*d*_5/2_ → 4*f* transition) at a step resolution of ≈0.2 eV. The magnetic information derived from the XMCD was related to the 7 *µ*_B_/Eu^2+^ ion moment.

Every experiment was thoroughly examined, with conclusions drawn from supporting data from further experiments. A definitive, trustworthy result depends critically on the quality of the samples. It is evident from these experimental results, using various approaches and high‐quality materials that interfacial magnetism exists on both the short‐ and long‐range scales up to ambient temperature.^[^ ^58^, ^81^ ^]^

## Conflict of Interest

The authors declare no conflict of interest.

## Author Contributions

F.K. and V.L. contributed equally to this work. The research was conceived and designed by F.K. and J.S.M. The samples were prepared and characterized by F.K. The high‐resolution TEM and Lorentz TEM experiments were carried out by L.S.B. and C.I.L.A. and analyzed by F.K.; the PNR experiments and data analysis were carried out by V.L.; the XAS/XMCD experiments and data analysis were carried out by F.K. and J.W.F.; the XRD experiments and data analysis were carried out by F.K. and H.Z.; the SQUID experiments and data analysis were carried out by F.K., M.E.J., and D.H.; and R.Y., A.M.C., and M.C.O. developed the micromagnetic model, performed numerical simulations, and analysis done by R.Y., A.M.C., M.C.O., and F.K. The data were interpreted by F.K., V.L., M.C.O., and J.S.M. All authors discussed the results and commented on the manuscript. The manuscript was written by F.K., V.L., and M.C.O. with contribution from all authors.

## Supporting information



Supporting Information

## Data Availability

The data that support the findings of this study are available from the corresponding author upon reasonable request.
