# Supplementary material for: Interface‐Induced Stability of Nontrivial Topological Spin Textures: Unveiling Room‐Temperature Hopfions and Skyrmions
Source: Adv Mater. 2025 Aug 18;38(1):e11754. doi: 10.1002/adma.202511754 (PMC12759252; doi:10.1002/adma.202511754)
Supplement: Supplementary file 1 — Supporting Information [file ADMA-38-e11754-s001.pdf]

ADVANCED MATERIALS

Supporting Information

for *Adv. Mater.*, DOI 10.1002/adma.202511754

# Interface-Induced Stability of Nontrivial Topological Spin Textures: Unveiling Room-Temperature Hopfions and Skyrmions

*Ferhat Katmis*, Valeria Lauter*, Rawana Yagan, Iuri S. Brandt, Arash M. Cheghabouri, Hua Zhou, John W. Freeland, Clodoaldo I. L. de Araujo, Michelle E. Jamer, Don Heiman, Mehmet C. Onbasli* and Jagadeesh S. Moodera**

Supporting Information

**Title: Interface-Induced Stability of Nontrivial Topological Spin Textures: Unveiling Room-Temperature Hopfions and Skyrmions**

*Ferhat Katmis*[*,†], *Valeria Lauter*[*,†], *Rawana Yagan, Luri S. Brandt, Arash M. Cheghabouri, Hua Zhou, John W. Freeland, Clodoaldo I. L. de Araujo, Michelle E. Jamer, Don Heiman, Mehmet C. Onbasli*[*], *& Jagadeesh S. Moodera*[*]

F. Katmis, J. S. Moodera

Department of Physics, Massachusetts Institute of Technology, Cambridge, MA-02139, USA

E-mail: katmis@mit.edu, moodera@mit.edu

F. Katmis, D. Heiman, J. S. Moodera

Francis Bitter Magnet Laboratory & Plasma Science and Fusion Center, Massachusetts Institute of Technology, Cambridge, MA-02139, USA

V. Lauter

Neutron Scattering Division, Neutron Sciences Directorate, Oak Ridge National Laboratory, Oak Ridge, TN-37831, USA

E-mail: lauterv@ornl.gov

R. Yagan, A. M. Cheghabouri, M. C. Onbasli

Department of Electrical and Electronics Engineering, Koç University, Istanbul, 34450, Türkiye

E-mail: monbasli@ku.edu.tr

L. S. Brandt, C. I.L. de Araujo

Departamento de Física, Universidade Federal de Viçosa, Viçosa, 36570-900, Brazil

H. Zhou, J. W. Freeland
Advanced Photon Source, Argonne National Laboratory, Argonne, IL-60439, USA

M. E. Jamer
Physics Department, United States Naval Academy, Annapolis, MD 21402, USA

D. Heiman
Department of Physics, Northeastern University, Boston, MA 02115, USA

M. C. Onbasli
Department of Physics, Koç University, Istanbul, 34450, Türkiye

## Supplementary Figures

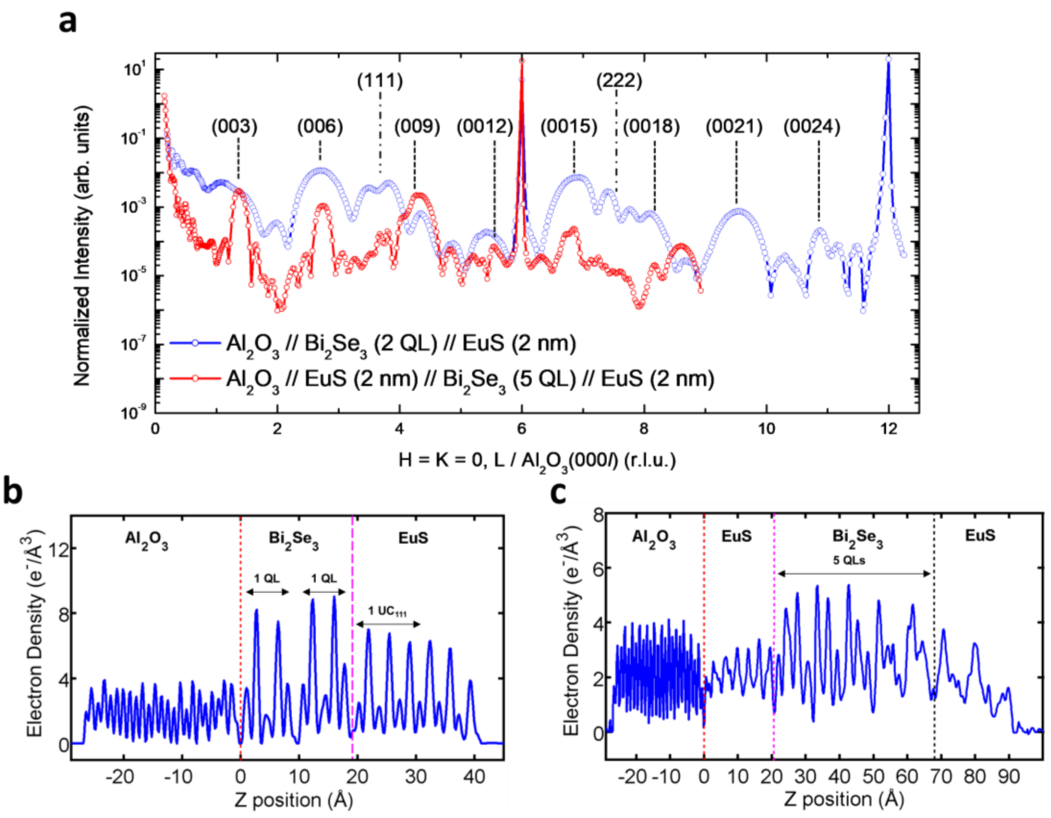


**Figure S1.** X-ray diffraction analysis for epitaxial bi- and tri-layer films grown on sapphire. **a**, In order to shed light on the interface microstructure we performed sets of crystal-truncation rod (CTR) measurements and employed coherent Bragg rod analysis (COBRA) to determine the real space electron density profile across the interfacial region with atomic precision.[1] The CTR measurements were performed with a six-circle diffractometer. The specular CTR (00*L*) of the bilayer and trilayer samples configuration were analysed by the COBRA method. From the density profile, **b**, for bilayer and **c**, trilayer, the interface between EuS and $Bi_2Se_3$ layers are well defined either in bilayer or trilayer. Sharp interface transition is shown without any chemical inter-diffusion by resolving the interface bond, clearly. Se atoms are in direct contact with the last oxygen layer of the sapphire. The $Bi_2Se_3$ is terminated with the Se layer at the interface and the S layer is in direct stacking with the Se layer on top of it via interfacial bonding separation by ~2.45 Å. Overall, EuS (111) on $Bi_2Se_3$–Sapphire shows a good epitaxial cube-on-hexagon growth relation.

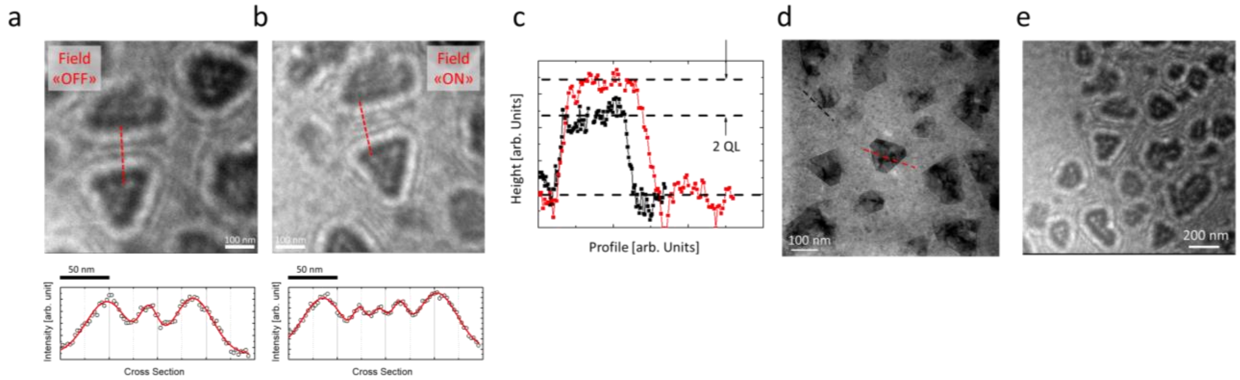


**Figure S2. a,** Hopfion and spin density wave formation. There is a discernible periodicity in the excitation between two separate island spin density waves when the field is off. **b**, A perpendicular field causes both the hopfion diameter and the wave periodicity to decrease. It also shows the potential pattern of interference. The line cuts are shown below for each. For the "Field-ON" configuration, the applied out-of-plane component of the magnetic field is 1.53 T. **c**, Height profile obtained from the line cut indicated by red and black dashed lines in **d**, revealing the characteristic truncated island morphology of epitaxially grown $Bi_2Se_3$ with thickness variations of ~2 QL between adjacent islands. **d**, Top-view TEM image showing the surface morphology of the trilayer layer film, with visible island formations characteristic of van der Waals material growth. **e**, LTEM image, displaying magnetic contrast that remains consistent across islands of varying thickness, demonstrating that the observed magnetic phenomena are intrinsic to the EuS / $Bi_2Se_3$ / EuS heterostructure.

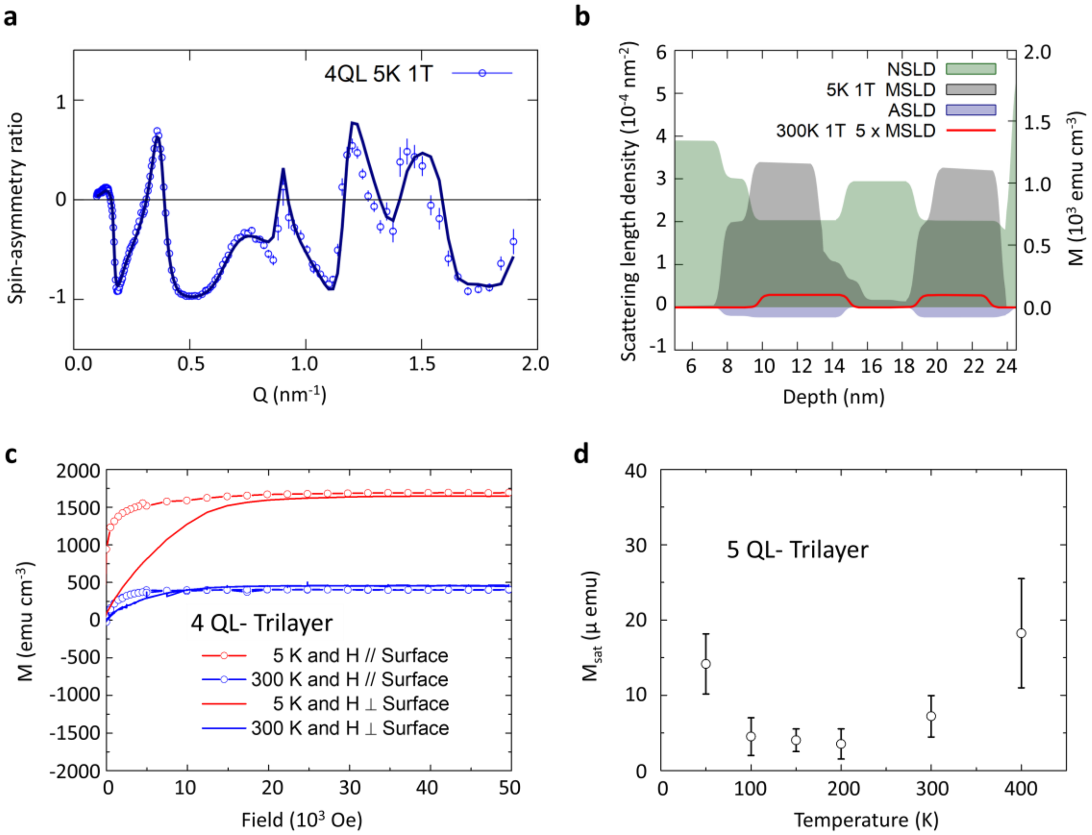


**Figure S3.** PNR and SQUID magnetometry measurements for trilayer EuS–$Bi_2Se_3$–EuS, PNR results for 4 QL-trilayer (EuS (5 nm) – $Bi_2Se_3$ (4 QL) – EuS (5 nm)) at 5 K in comparison with 300 K data. In **a**, the corresponding spin-asymmetry (SA) ratio and model fits are displayed with solid lines, SA = $(R^+-R^-)/(R^++R^-)$, is derived from the reflectivity measurement fitting for 4 QL-trilayer sample. In **b**, neutron nuclear (NSLD, green), magnetic (MSLD, grey) and absorption (ASLD, purple) scattering length density profiles are shown along the trilayer portion of the epitaxial sample which were recorded at 5 and 300 K with an in-plane 1 T field. In **c**, measurements of M(H) at 5 and 300 K temperature in a parallel and perpendicular field configurations for 4 QL-trilayer (EuS (5 nm) – $Bi_2Se_3$ (4 QL) – EuS (5 nm)) sample. Compared to the sample shown in Figure 3, this one is distinct. This has a couple orders of magnitude larger saturation moment at 300 K, however the low temperature values is 25% lower. In **d**, the saturation moment versus temperature data is shown for 5 QL-trilayer sample.

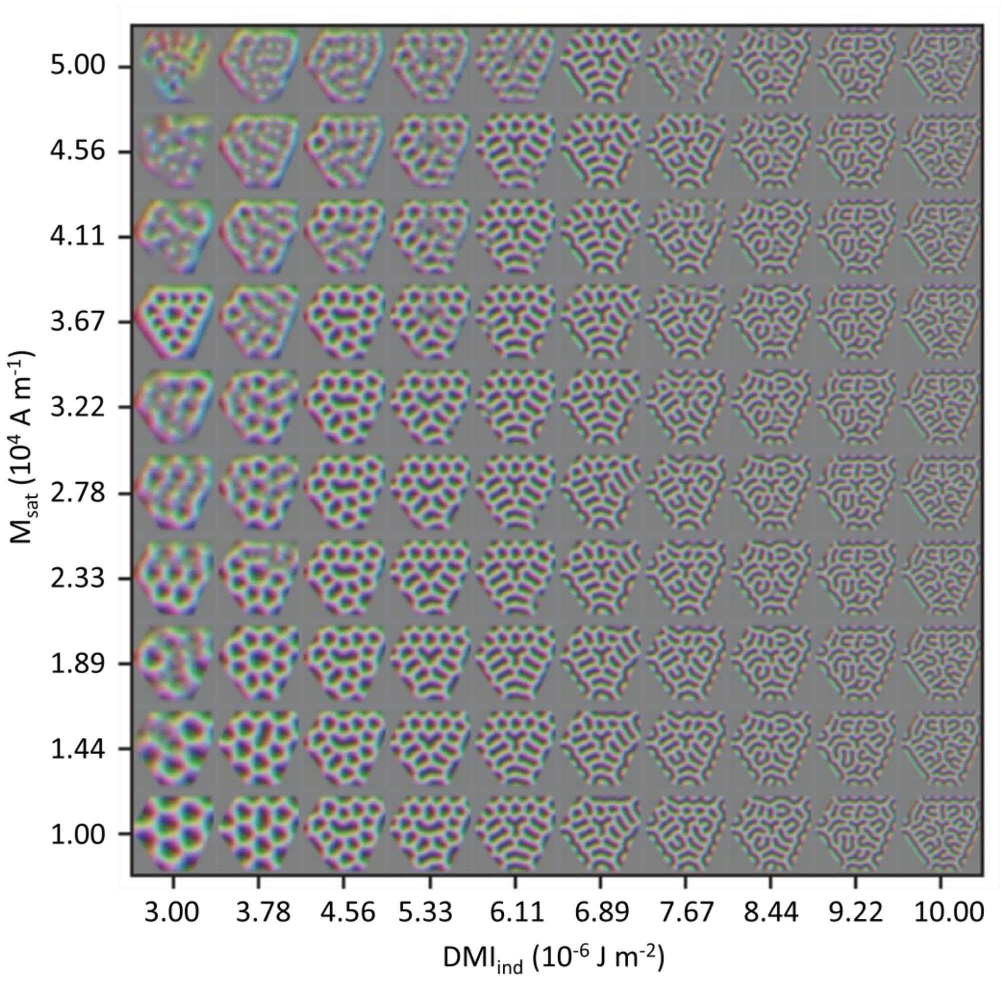


**Figure S4.** Micro Magnetic Modelling without hopfion, the effect of change in the DMI and saturation magnetization on the stabilization of a single skyrmion island without hopfion ring.

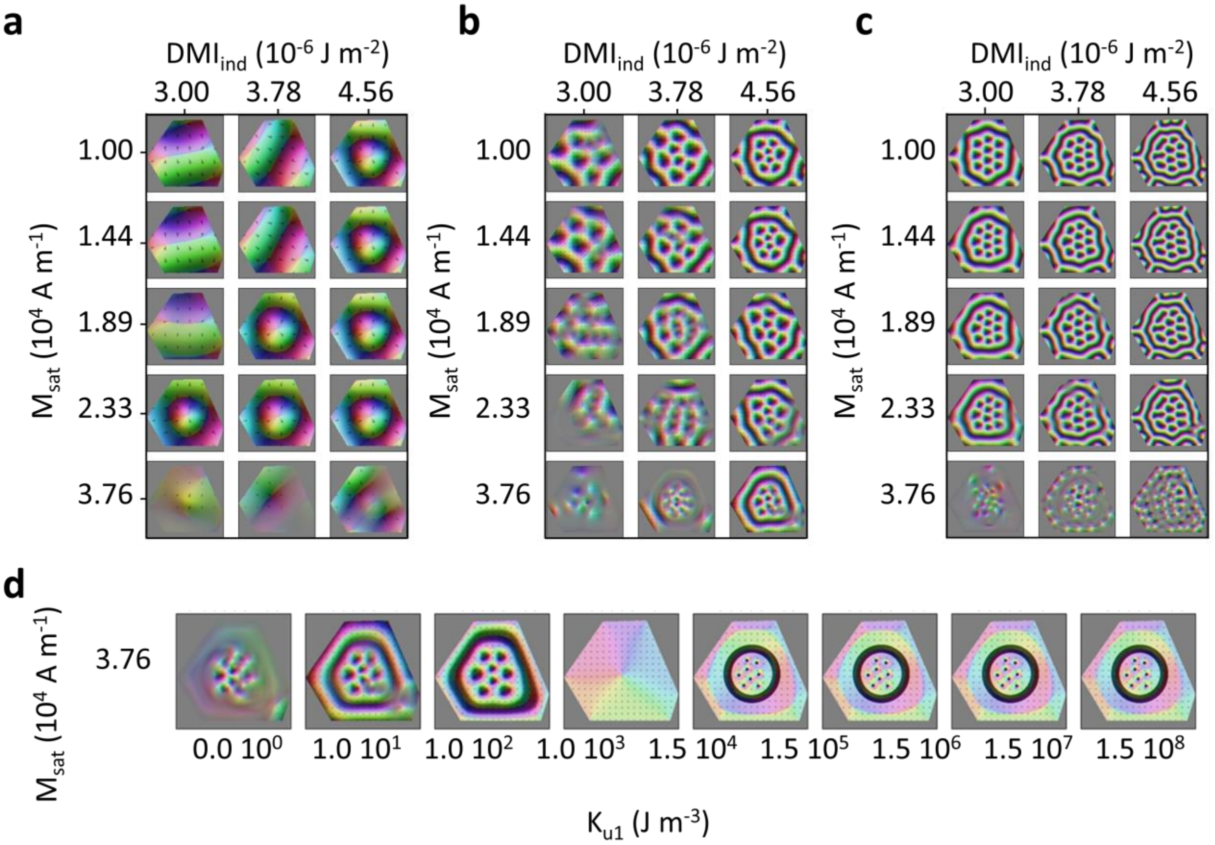


**Figure S5.** Micromagnetic simulations of magnetic texture with hopfion rings for trilayer films. The corresponding simulations for Figure 2a were run on irregular hexagon geometry with sizes at 100 in **a**, 300 in **b**, and 500 nm$^2$ in **c**. The entire isosurfaces of the skyrmion and hopfion lattice construction are matched with experimental data in **b**, due to their accurate size and shape by convenient $M_{sat}$ and *DMI* parameters. In **d**, a large range was also run for the corresponding uniaxial anisotropy parameters $K_{u1}$ with $DMI_{ind} = 4.56 \times 10^{-6}$ J m$^{-2}$, $A_{ex} = 1.94 \times 10^{-14}$ J m$^{-1}$, and $M_{sat} = 37.6$ kA m$^{-1}$ with regular triangle geometry with sizes at 300 nm$^2$.

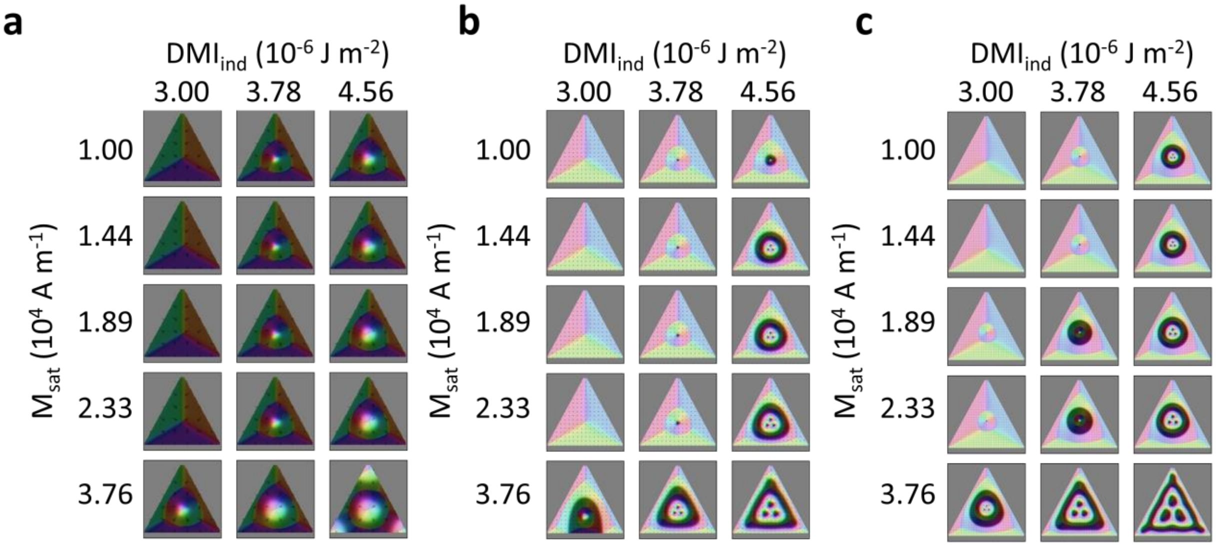


**Figure S6.** Micromagnetic simulations of 3 skyrmions with hopfion ring formation for trilayer films. The corresponding simulations for Figure 2g were run on regular triangle geometry, which includes 3 skyrmions, with sizes at 100 in **a**, 300 in **b**, and 500 $nm^2$ in **c**. The entire isosurfaces of the skyrmion and hopfion lattice construction are matched with experimental data in **b**, due to their accurate size and shape by convenient $M_{sat}$ and *DMI* parameters. A large range was also run for the corresponding uniaxial parameters with $DMI = 4.56 \times 10^{-6}$ J $m^{-2}$, $A_{ex} = 1.94 \times 10^{-14}$ J $m^{-1}$, and $M_{sat} = 37.6$ kA $m^{-1}$.

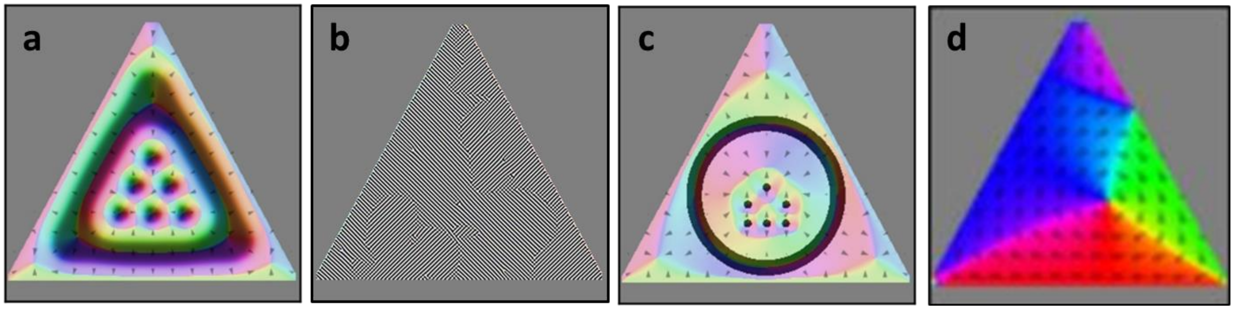


**Figure S7.** Micromagnetic model on extended parameters for key material parameters. The effect of increasing a few orders of magnitude of the key material parameters in our study from those in the initial model shown in **a**, where we obtain *DMI* = 4.56 μJ $m^{-2}$, $M_{sat}$ = 37.6 kA $m^{-1}$, and $K_{u1} = 1\times10^{3}$ ($A_{ex}$ is fixed in all figures at $1.94\times10^{-14}$ J $m^{-1}$). Increasing the interfacial *DMI* strength to 1 mJ $m^{-2}$ converts the skyrmion lattice and hopfion ring into a magnetization state of a strip-like domain as we allow them to relax in the micromagnetic model as shown in **b**. In **c**, having a high anisotropy constant, $10^{5}$ J $m^{-3}$, a couple of orders of magnitude larger prevents the initial magnetization state from relaxing, and we observe the high symmetry shapes of the skyrmion lattice and hopfion ring. The strong perpendicular uniaxial anisotropy field inhibits the moments from canting or reorienting themselves and overpowers the shape anisotropy effects. Having an order of magnitude larger $M_{sat}$, 376 kA $m^{-1}$, in **d**, increases the demagnetization field effect (allows for large domain formation) and shape anisotropy, dissolving the magnetic features in the ferromagnetic layers.

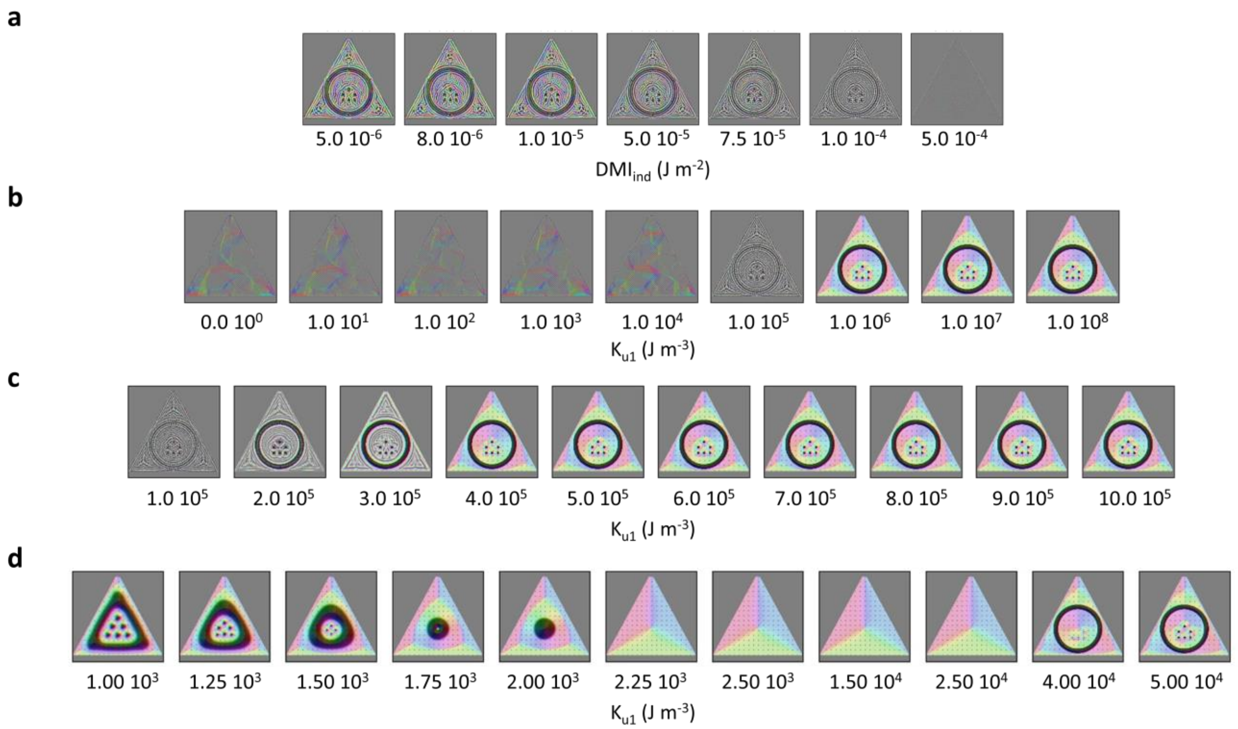


**Figure S8.** Micromagnetic model on extended parameters. In **a,** the effect of introducing the *DMI* constant between 5.0 μJ $m^{-2}$ to 50 mJ $m^{-2}$ for the magnetic features with high saturation magnetization $M_{sat}$ = 800 kA $m^{-1}$ and high uniaxial anisotropy $K_{u1} = 1\times10^5$ J $m^{-3}$ are shown. High $M_{sat}$ increases the magnetostatic interactions and the demagnetization energy that induces strip-like domain formation. Increasing the *DMI* allows the strip-like domain system to dominate around these features and eventually converts the ring and skyrmion lattice into a system of such domains. In **b**, results show that keeping $M_{sat}$ and *DMI* high at 800 kA $m^{-1}$ and 10 mJ $m^{-2}$, respectively, while increasing the uniaxial anisotropy constant restores the ring and skyrmion lattice initial magnetization states, and the strip-like domain system disappears, which also shown for a finer sweep of $K_{u1}$ in **c**. In **d**, finer sweep for the region at the vicinity of $K_{u1}$=1.5 x $10^4$ J $m^{-3}$ with $DMI = 4.56 \times 10^{-6}$ J $m^{-2}$, $A_{ex} = 1.94 \times 10^{-14}$ J $m^{-1}$, and $M_{sat}$ = 37.6 kA $m^{-1}$ with regular triangle geometry with sizes at 300 $nm^2$.

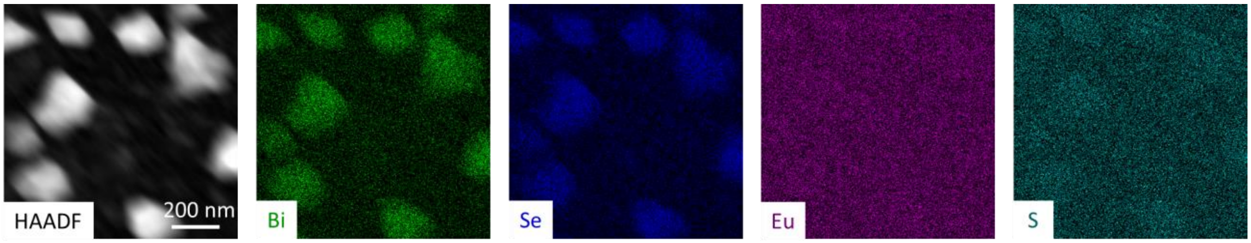


**Figure S9.** HAADF and EDS measurements for trilayer samples grown on a grid. Energy dispersive spectroscopy (EDS) map displaying the spatial atomic distribution of a trilayer sample grown on $Si_3N_4$ membrane. The elements Bi, Se, Eu, and S are evenly dispersed throughout the film surface, based on the element mapping pictures. The element molar ratios of Eu:S and Bi:Se are around 1:1 and 2:3, respectively, as anticipated for grown materials. Because of their lower packing ratio, crystallite areas contrast less than polycrystalline regions. The presence of distinct domain sizes is validated using high-angle annular dark field (HAADF) pictures, in which the contrast of the images is approximately correlated with the square of the atomic number of the chemical species under investigation. The temperature of deposition affects both the domain size distribution and their inter-domain spaces. At low temperatures, the domain sizes decrease down to 2–10 nm; nevertheless, at high temperatures, the situation drastically shifts and causes a notable rise up to a few of hundred nm. The sharp rise in crystalline sizes suggests that either larger domains are directly growing, or tiny domains are coalescing because of the species' greater mobility at higher temperatures.

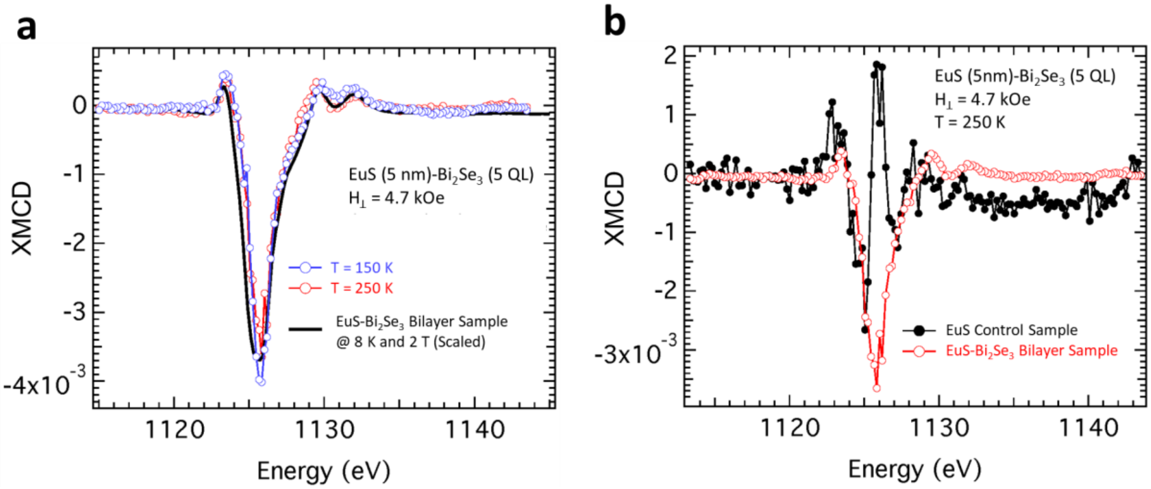


**Figure S10.** High-Temperature XMCD data analysis for EuS-$Bi_2Se_3$ interface. The XMCD (X-ray Magnetic Circular Dichroism) spectra as a function of photon energy for $Eu^{2+}$ state is shown, where the spectra are taken as a function of temperature to get magnetic features of Eu atoms after aligning the moments by 5 T applied field for both EuS (5 nm) – Sapphire in **b** and EuS (5 nm) – $Bi_2Se_3$ (5 QL) – Sapphire samples in **a** and **b**. The depicted scans were evaluated with X-ray energy over Eu $M_5$-edge (~1128 eV, $3d_{5/2} \rightarrow 4f$ transition) using ~0.2 eV step resolution and averaged out from several scans. From the temperature dependence measurements, according to Curie-Weiss at 250 K, it is expected to have 60% of the 150 K value, however, at 250 K, we observed 83% of the 150 K value where similar behaviour was observed with SQUID magnetometry measurements.[2] Given that the control sample's signal in **b** deviates from Eu's line shape, it is most likely a derivative artifact from sample charging.

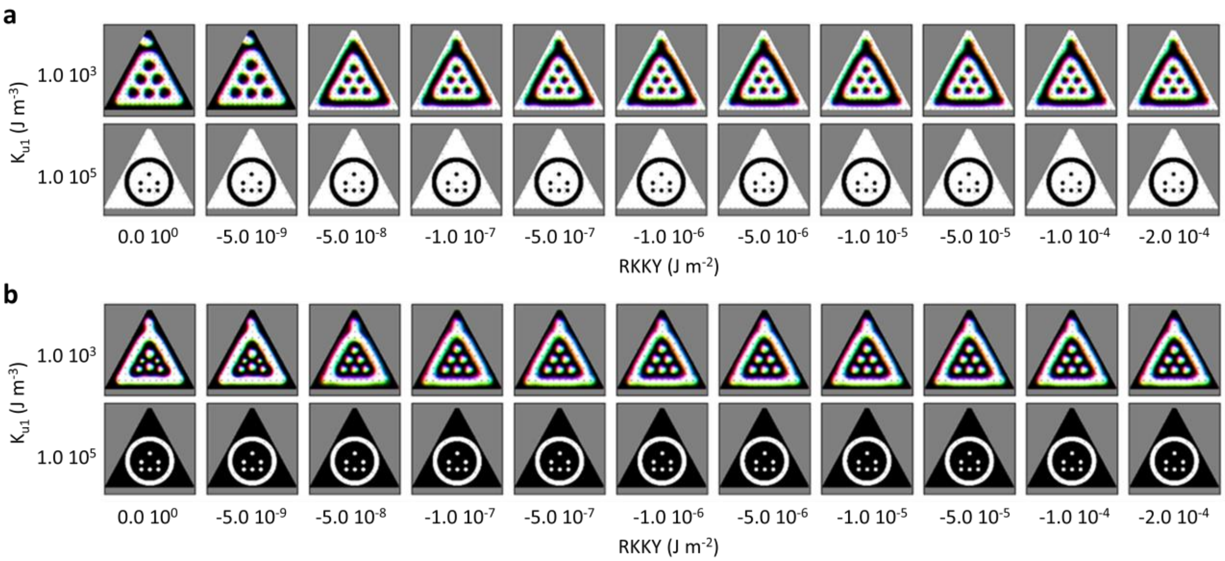


**Figure S11.** Micromagnetic model for interlayer exchange coupling and RKKY for trilayer system. In **a**, the bottom layer and in **b** the top layer z-slice image of the skyrmion lattice simulated results are shown. Here, we introduced the antiferromagnetic coupling (AFM) between the top and bottom ferromagnetic layers of EuS. The Ruderman-Kittel-Kasuya-Yosida (RKKY) constant was swept for the range shown Figures **a**, and **b** for two values of out-of-plane uniaxial anisotropy constant $K_{u1}$. The effect was mediated through the scaled exchange constant between the top and bottom interfaces that were initialized with a Neel skyrmion lattice and a hopfion ring with z-plane mirrored magnetization profiles. The rest of the material parameters used in these runs were as before. The RKKY constant was varied from 0 to -0.2 mJ $m^{-2}$, where the strength of AFM coupling can be a function of the spacer layer thickness[3] and can act proportional to the TI layer thickness.[4]

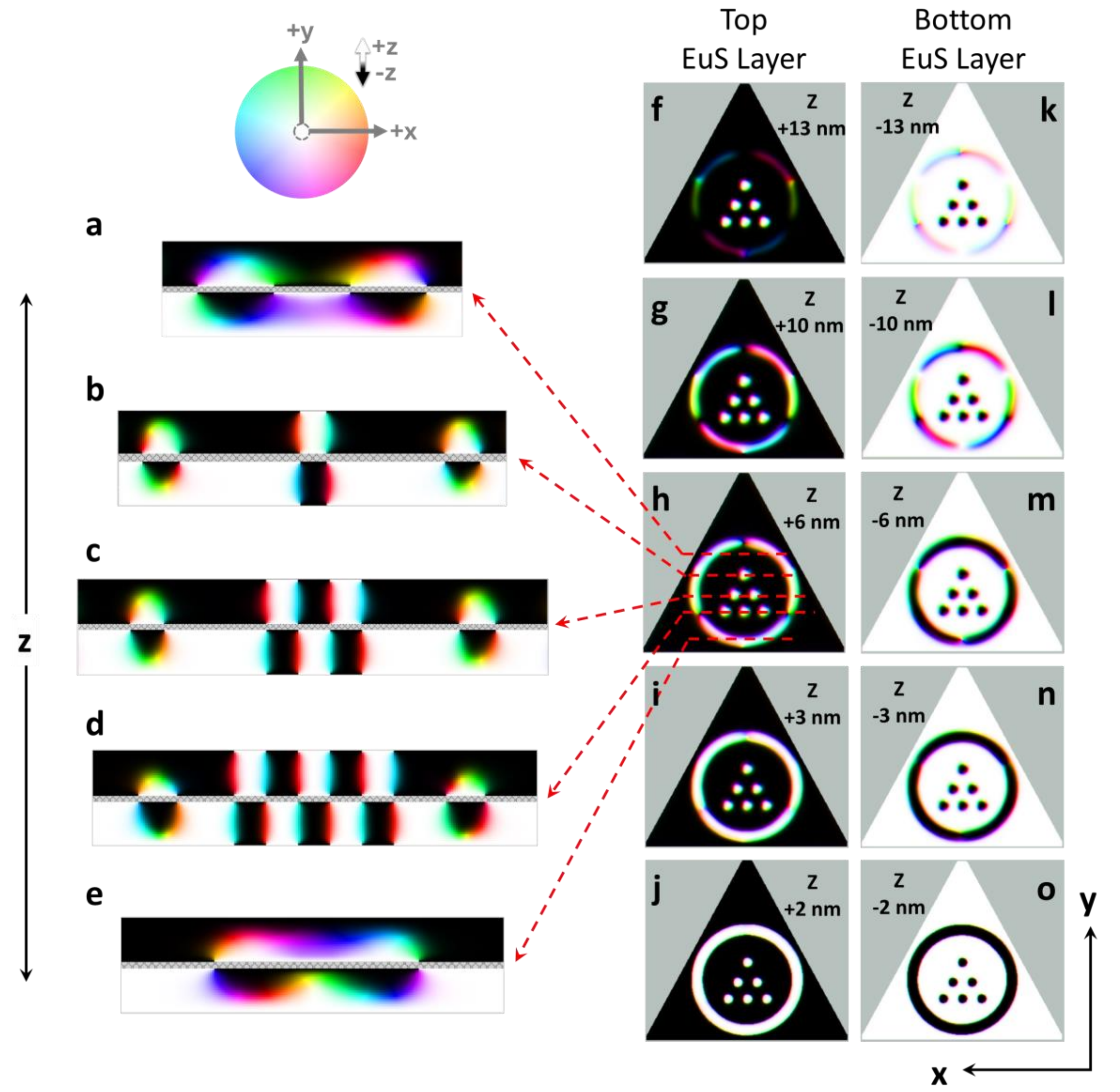


**Figure S12.** Extended micromagnetic simulation across the entire thicker trilayer heterostructure. The micromagnetic simulation of EuS (20 nm) / $Bi_2Se_3$ (5 QL) / EuS (20 nm) shows a 3D representation across the thickness of each EuS layer. The simulation illustrates the skyrmion lattice surrounded by the hopfion in the antiferromagnetically coupled EuS layers through $Bi_2Se_3$ layer, which is not explicitly included in the simulations and is marked as a grey-crossed area. The phase profile along x-z view at different y positions is shown in **a** to **e,** while the lateral view is depicted in the x-y plane in **f** to **o**. Lateral cross-sections were taken at different EuS thicknesses, specifically at ±2, ±3, ±6, ±10, and ±13 nm, corresponding to their distances from each $Bi_2Se_3$ interface, where + and - signs indicate for the top and bottom EuS

layers, respectively. The color bar shows the spin phase changes for increasing azimuthal and polar angles.

## References:

[1] Y. Yacoby, M. Sowwan, E. Stern, J. O. Cross, D. Brewe, R. Pindak, J. Pitney, E. M. Dufresne, R. Clarke, *Nature Materials* **2002**, 1, 99.

[2] F. Katmis, V. Lauter, F. S. Nogueira, B. A. Assaf, M. E. Jamer, P. Wei, B. Satpati, J. W. Freeland, I. Eremin, D. Heiman, P. Jarillo-Herrero, J. S. Moodera, *Nature* **2016**, 533, 513.

[3] T. McKinnon, B. Heinrich, E. Girt, *Physical Review B* **2021**, 104.

[4] M. Li, W. Cui, J. Yu, Z. Dai, Z. Wang, F. Katmis, W. Guo, J. Moodera, *Physical Review B* **2015**, 91, 014427.
